# Primary endodermal sinus tumor originating from the sacral ligament: a case report and review of the literature

**DOI:** 10.1186/s12884-023-05849-2

**Published:** 2023-07-20

**Authors:** Han Lu, Dongsong Jia, Qionglan Tang, Shanrong Shu

**Affiliations:** 1 Department of Gynecology and Obstetrics, The First Affiliated Hospital of Jinan University Guangzhou, Guangzhou, 510630 Guangdong People’s Republic of China; 2 Department of Pathology, Sun Yat-sen Memorial Hospital, Sun Yat-sen University, Guangzhou, 510120 People’s Republic of China

**Keywords:** Endodermal sinus tumor, Sacral ligament, Postoperative pathology

## Abstract

**Background:**

Endodermal sinus tumor (EST) is a malignant tumor originating from the ovary or testis. In most case, ultrasound examination shows ovarian mass. But there is a special kind of extra-gonadal endodermal sinus tumor, which occur in organs other than gonads with insidious onset. Here we reported a case of endodermal sinus tumor, which originated from the sacral ligament presenting as an acute lower abdominal pain.

**Case presentation:**

A 14-year-old girl was admitted to the hospital because of acute lower abdominal pain. The ultrasound showed a mass with 72 mm × 64 mm × 50 mm in Douglas, and there was no abnormality in bilateral ovaries and fallopian tubes. Laparoscopic exploration showed a large amount of blood clots in the pelvic cavity. After removal of the blood, we found rotten fish-like tissue in the left sacral ligament, rapid pathology suggested endodermal sinus tumor. After the operation, we retrospectively examined the value of alpha-fetoprotein (AFP), which was found to be elevated, and post-operative paraffin pathology confirmed the diagnosis. After four cycles of BEP chemotherapy, exploratory laparotomy was performed to remove the visible lesion, but postoperative pathology showed no abnormality. At the one-year follow-up, the patient remained recurrence-free.

**Conclusion:**

Extra-gonadal germ cell tumors are rarely reported. When young teenagers complain of acute lower abdominal pain with elevated AFP, but there was no lesion in bilateral ovaries and fallopian tubes, we must think about the possibility of endodermal sinus tumors. Accurate diagnosis facilitates complete resection of lesions and improves patient’s outcomes.

## Introduction

Endodermal sinus tumor (EST), also known as yolk sac tumor (YST), is a typical germ cell tumor (GCT), which can occur in the gonads or extra-genital glands. Germ cell tumors are more common in the testes and ovaries. Extra-gonadal germ cell tumors are very rare, with an overall incidence of 1.8 to 3.4 per 1 million in the United States [1]. In China, the incidence of EST ranks the first among ovarian malignant germ cell tumors, and about 1/3 of patients are diagnosed before menstruation. EST have a high incidence at the age of 1–35 years, with insidious onset (especially extra-gonadal tumors), rapid progression, susceptibility to metastasis, and poor prognosis [2]. Extra-ovarian EST is a rarely seen in pelvic localization. Here we reported a case of EST, which occurred in the sacral ligament.

## Case presentation

**Fig. 1:**
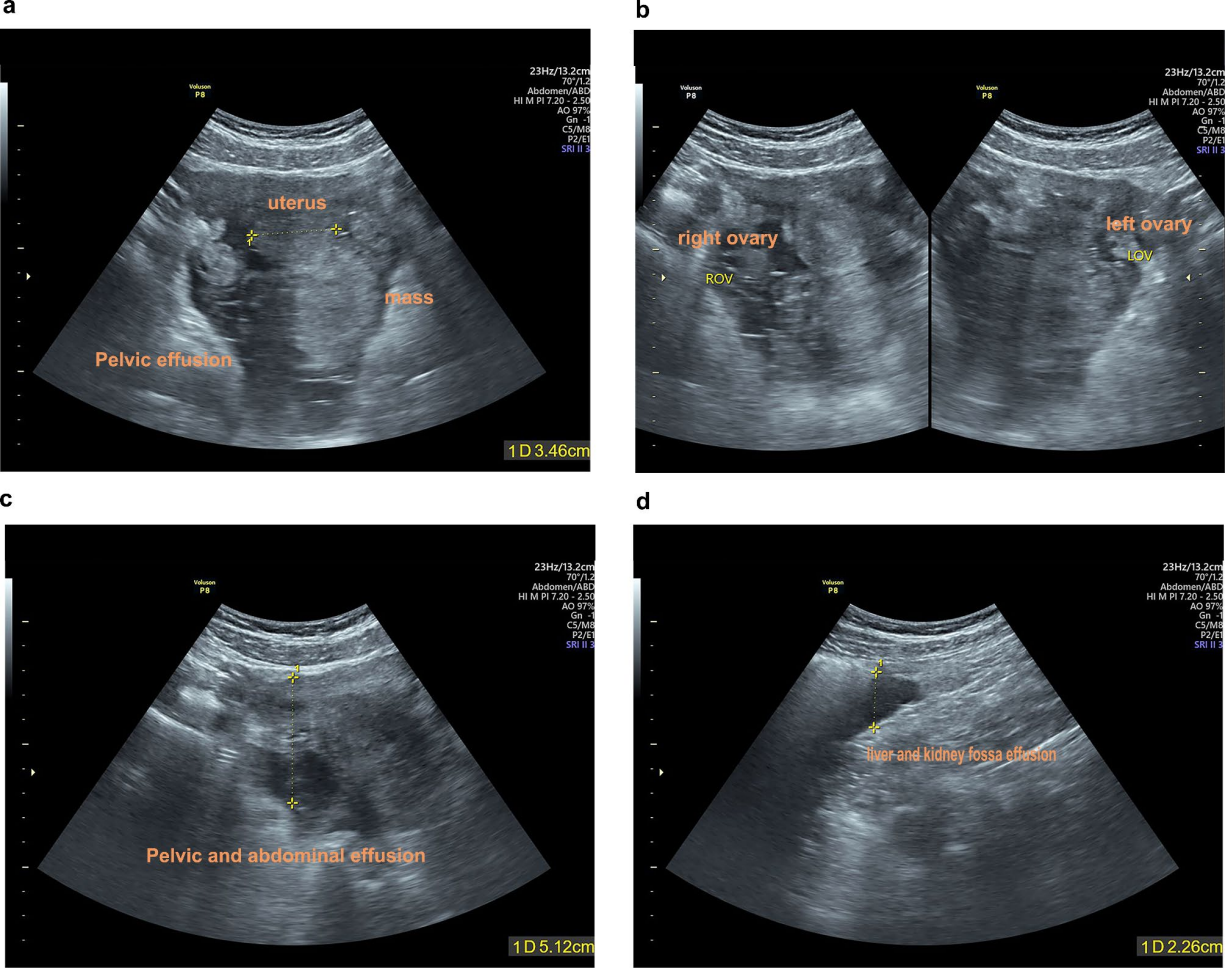
Ultrasound Imaging before operation. **(a)** An uneven slightly higher echo photophore is found at the back of the uterus and is surrounded by a large volume of fluid. **(b)** Normal morphology of the left and right ovary. **(c)** Pelvic and abdominal effusion. **(d)** Liver and kidney fossa effusion

A 14 years old girl was admitted in our hospital complaining with severe lower abdominal pain accompanied with nausea and vomit, which occurred during dinner. Menarche is 11 years old and the menstrual cycle is irregular. Gynecological examination was not performed because the girl was not sexually active. Physical examination showed mild tenderness and rebound tenderness. General hematological parameters such as blood routine and electrolyte check were normal. Ultrasonography showed the bilateral ovaries and fallopian tubes was normal, but an uneven slightly higher echo photophore was found at the back of the uterus, the size was about 72 mm × 64 mm × 50 mm, the boundary was unclear, the morphology was irregular. But the boundary of the ovaries was very clear. Meanwhile, we found a lot of fluid in liver and kidney fossa and bilateral iliac fossa (Fig. 1).

**Fig. 2:**
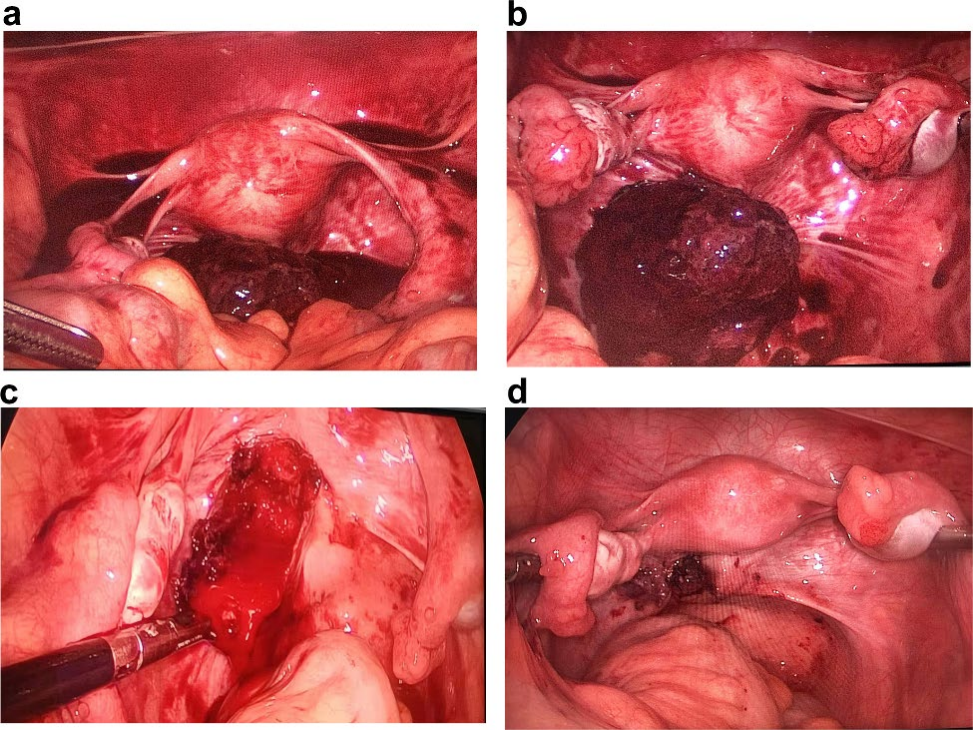
Intraoperative images. **(a)** Exposure of the uterus, the bilateral ovaries and fallopian tubes and abdominal blood. **(b)** An approximately 8 cm mass with irregular morphology, uneven surface, dark red clot and ruptured capsular is exposed. **(c)** The lesion was originated from the left sacral ligament. **(d)** The image after removal of the lesion

Considering relief of the pain and the possibility of intra-abdominal bleeding, we performed laparoscopy. During the operation, we found liquid blood about 500 ml in the abdominal cavity (Fig. 2a). After sucking those blood, we saw a solid mass about 8 cm in size with irregular morphology, uneven surface, dark red clot and ruptured capsular (Fig. 2b). Bilateral fallopian tubes and ovaries were normal. At last, we found the lesion was next to the left sacral ligament (Fig. 2c).

**Fig. 3:**
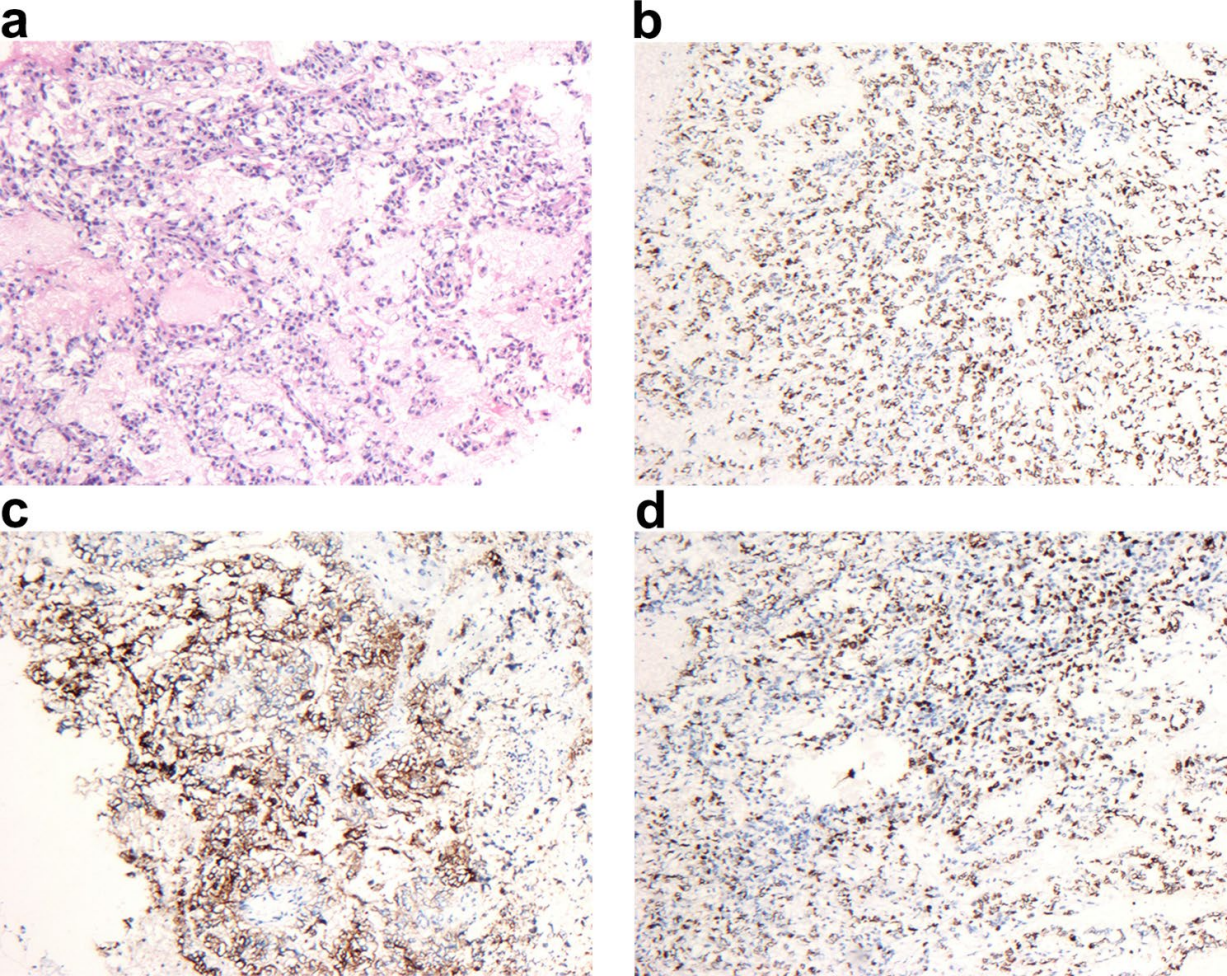
Light microscopic appearance and immunohistochemical staining of EST originating from the left sacral ligament. **(a)** Endodermal sinus tumor (H and E, 1 × 40). **(b)** Glypiacan-3 (+). **(c)** AFP (+). **d.**ki67 (+)

When removing the mass, we detected some rotten fish fleshy tissue (Fig. 2d). Rapid pathological examination suggested germ cell tumors with the possibility of endodermal sinus tumor. We retrospectively detected the value of AFP, which was 1162.3 ng/ml. Postoperative paraffin pathology verified the diagnosis with AFP (+), SALL4 (+), Glypican-3 (+), PLAP (-), CDX2 (partial +), CD117 (-), CK7 (-), Calretinin (-), ki67 about 80% (+) (Fig. 3).

**Fig. 4:**
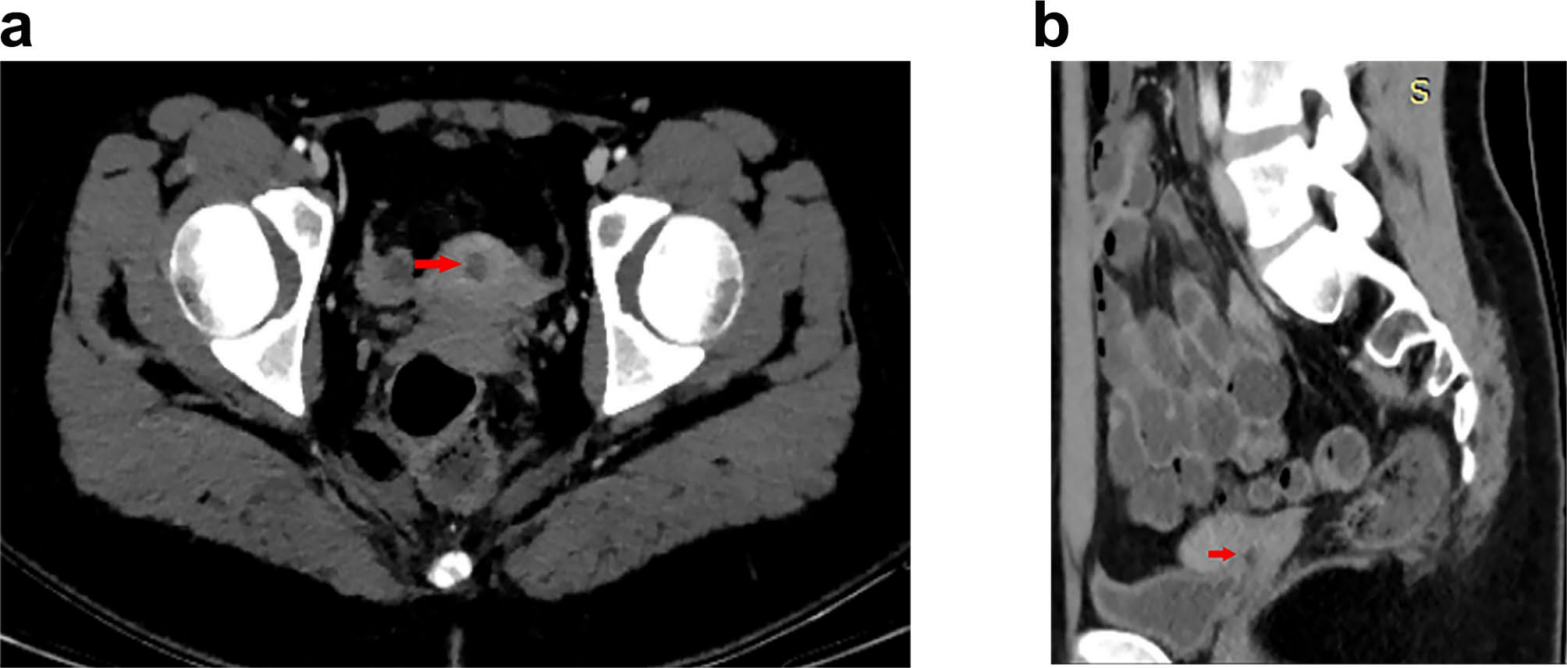
Pelvic CT: a lesion about 1 cm in the douglas sac

Twenty-two days after the laparoscopy, this girl experienced 4 courses of BEP chemotherapy. At the end of the third chemotherapy, we found AFP decreased to 6.5 ng/ml. Two months after the last chemotherapy, the patient underwent CT examination, which showed a lesion about 1 cm in the douglas sac (Fig. 4) and no remote metastasis. Considering the patient was a younger girl, and the possibility of existence of residual tumor, we performed exploratory laparotomy, and fortunately, the mass showed in the CT was inflammation hyperplasia. We followed the patient by examining the AFP and whole abdominal MRI for one year, and found no symptom of recurrence.

## Discussion and conclusions

90% of GCT are found in the gonad and 10% of case existed in different extra-gonadal sites. Moreover, the most common extra-ovarian sites of EST are mediastinum, vagina, brain and retroperitoneum [3]. EST originating from the endometrium or broad ligament have been reported in previous article, but these extra-gonadal EST are very rare as in our case [4]. When the tumor grows to a certain size, different clinical manifestations may appear according to the tumor site, mostly pain or masses, which are not specific. Endodermal sinus tumors are prone to hematogenous metastases in early stages, and most patients are already in advanced stages when they are diagnosed with vascular invasion or metastases that are difficult to remove cleanly by surgery, thus patients usually have a poor prognosis [5].

Serum AFP measurement has an important role in the diagnosis of EST, assessment of efficacy and monitoring of recurrence or metastasis [3, 6].On ultrasound examination, malignant ovarian EST tend to be unilateral, large, multifoveal solid or solid, with finely textured tissue, slightly hyperechoic, and richly vascularized [7]. On MRI, EST may show areas of hemorrhage and are valuable in assessing the presence of metastases to the lymph nodes, greater omentum, lung, liver, or bone [8]. Recently, Li et al. [9, 10] investigated CT images of ovarian EST, extra-gonadal EST and other ovarian malignancies the result showed that most of the ovarian endodermal sinus tumors appearanced large, well-defined solid-cystic masses, intra-tumor hemorrhage, marked heterogeneous enhancement, and enlarged intratumoral vessels. Among them, the intratumoral vascular enlargement with diagnostic specificity was also called “bright dot sign”.

However, the diagnosis still depends on pathological examination. Typical histological features include reticular microcystic areas with hyaline globules and amorphous acellular basement membrane material [11]. Among the recognized histologic patterns yolk sac tumors are considered to usually exhibit a combination of 2 or more of the following structural patterns: microcystic/reticular, papillary, solid, festoon, polyvesicular-vitelline, glandular pattern, and hepatoid. Typical Schiller-Duval vesicles can be seen. The typical immunophenotype is positive for immunoreactivity for AFP, Glypican 3 and SALL4. Notably, AFP is very specific for the diagnosis of EST, but is not sufficiently sensitive. Glypican 3 and SALL4 are sensitive markers in EST, but are insufficiently specific and a differential diagnosis that needs special mention is clear cell carcinoma [12, 13]. In our case, AFP, SALL4 and Glypican-3 were all strongly positive, which was consistent with literature reports.

Extra-glandular germ cell tumors are rare and are found in most structures along the midline, with the probability of 46% to experience of occurrence in the brain. The mechanism of extra-gonadal EST is still not well defined. Nowadays, there are two main hypotheses about the causes of extra-gonadal germ cell tumors. The first hypothesis maintains that the anomaly in somatic cells differentiation is responsible for the origin of the tumor. This could be a possible explanation for the occurrence of yolk sac tumors in stomach, endometrium or lung [14]. The second hypothesis proposes that extra-gonadal germ cell tumors originate from primitive germ cells which undergo malignant transformation during the first trimester of pregnancy. These cells may either come from outside of the gonads or spread to other areas from the gonads. These tumors are commonly found in areas such as the retroperitoneum, sacrococcygeal region, mediastinum, and brain, which may arise from germ cells that were either blocked or misplaced during embryonic migration [15]. The second hypothesis is generally accepted as the cause of extra-gonadal germ cell tumors [16, 17]. McKenney et al., however, suggests that extra-gonadal tumors may correspond to the spread of an occult undiagnosed or regressive malignant lesion in the gonads [18]. This is also supported by the “regressive” features of the peripheral lesions with signs of scarring [19].

To date, no treatment guidelines have been developed for extra-gonadal YST because of the rarity of the disease. Previously, the conventional view was that the prognosis of EST was extremely poor, regardless of the primary site. In the last decade, the treatment paradigm of adjuvant chemotherapy after surgery has significantly improved the survival rate of patients. Vincristine, actinomycin D, cyclophosphamide and vincristine, bleomycin and cisplatin have been used to treat patients with ovarian and extra-ovarian EST [20, 21]. Recently, it is reported that patients with advanced germ cell have complete remission, and the cure rates is as high as 80–90% after treated with bleomycin, etoposide and cisplatin (BEP) chemotherapy [3, 22]. The ideal treatment strategy for EST is complete resection of the tumor accompanied with postoperative adjuvant chemotherapy [23]. There is no consensus on the role of systematic lymph node dissection in the treatment of EST. but ignoring staged laparotomy appears to increase the recurrence rate, although there is no effect on overall survival [24]. In advanced stages of EST, the goal of surgery should be to remove as much of the tumor as possible without lymphatic clearance. Because EST is highly sensitive to chemotherapy, surgery should minimize complications and lymph node dissection should be performed only in cases of abnormal lymph nodes, thus ensuring that chemotherapy can be administered as soon as possible [25]. For postoperative EST patients without or only little residual tumor, 3 courses of BEP is preferred, and for patients with large residual lesion, 4 courses of BEP are recommended [26]. In order to reduce the harmful effects of chemotherapy on the ovaries, the use of Gn-RH agonists to inhibit ovulatory activity during chemotherapy is suggested [27]. During the treatment, serum AFP changes were continuously monitored to assess the efficacy, and the decrease of serum AFP level indicated that the treatment was effective, and the increase of serum AFP level again might indicate tumor recurrence or drug resistance. Follow-up visits are performed every 3 months for the first 2 years after treatment, every 6 months for the third year, and then annually until progression of the disease [16].

Our case was a typical extra-gonadal germ cell tumors originated from the left sacral ligament with normal ovarian structure. Maybe, it results from the misplace during embryonic migration. The mass envelope was ruptured with bleeding, which may contribute to the patient’s abdominal pain. The mass was seen to be rotten fish-like with abundant blood flow. After complete resection of the mass, intraoperative rapid pathology suggested that the mass was suspicious for a malignant tumor of genital system. Regrettably, we did not resect the omentum and lymph nodes intraoperatively. Postoperatively, we diagnosed EST by paraffin pathology and the above mentioned tumor markers.

In general, in adolescent girls, pelvic masses occurring outside the uterus and bilateral adnexa mainly included endometriosis, inflammatory pelvic masses, and gastrointestinal malignancies. In our case, a girl without sex activity had a huge mass in the posterior part of the uterus, it is impossible to have endometriosis or pelvic inflammatory diseases. Therefore, we should have been alert to the possibility of malignancy tumor to test the tumor markers and performed imaging prior to surgery when possible. Unfortunately, we made a misdiagnosis for no testing of tumor markers, such as AFP and did not resect the omentum and lymph nodes during the operation. Fortunately, the tumor did not metastasize and we intactly removed the mass. Four courses of standardized chemotherapy were given after surgery, and the patient’s AFP decreased significantly after surgery. We found no recurrence during follow-ups. As mentioned above, less invasive surgery accompanied with standard chemotherapy seems to be the better treatment modality for patients with EST. In addition, in China, adolescent girls do not routinely undergo ultrasound examinations. Ultrasound examinations are performed only experiencing lower abdominal pain or abnormal vaginal bleeding. In this case, if the patient had not had lower abdominal pain, she would not have been able to detect a mass in her pelvis, let alone to detect a malignant tumor. Therefore, we should pay more attention to the routing medical examination for women especially for those who has family history of malignant. In conclusion, we must remind the existence of extra-gonadal germ cell tumors, especially for those teenagers who present with mass in pelvic, but no lesion in the ovary, which helps us to make appropriate treatment for them.

## Data Availability

Data supporting the results of this study are available from the corresponding author. Due to privacy or ethical restrictions, these data are not publicly available.

## Abbreviations

EST: Endodermal sinus tumor
YST: Yolk sac tumor
GCT: Germ cell tumor
AFP: Alpha-fetoprotein
